# Hospital-onset IgA vasculitis triggered by infectious endocarditis

**DOI:** 10.1016/j.idcr.2023.e01865

**Published:** 2023-07-28

**Authors:** Kento Furuya, Naoya Itoh

**Affiliations:** a Department of Clinical Laboratory Medicine, Shizuoka General Hospital, Aoi-ku, Shizuoka, Japan; b Division of Infectious Diseases, Aichi Cancer Center Hospital, Chikusa-ku, Nagoya, Japan

**Keywords:** Infectious endocarditis, Immunoglobulin A (IgA) vasculitis, *Staphylococcus aureus*

## Abstract

This is a case of IgA vasculitis developed in the hospital during treatment of infective endocarditis. When purpura appears in a patient under IE treatment, we should consider IgA vasculitis as a differential diagnosis and check renal function.

## Case presentation

**Fig. 1:**
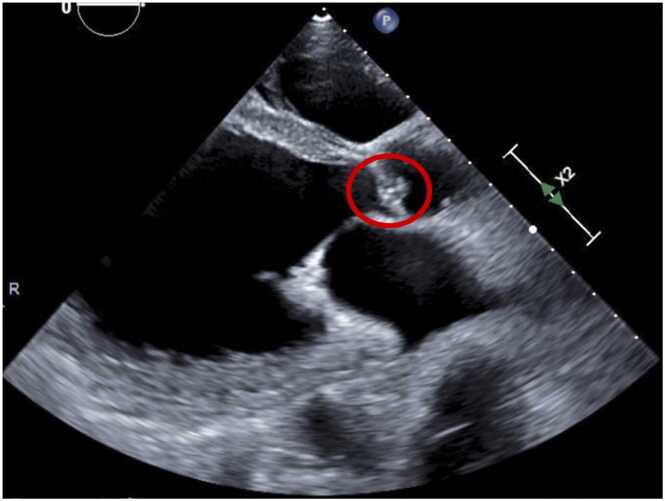
Transthoracic echocardiography showing a 5 × 4 mm vegetation on the aortic valve.

**Fig. 2:**
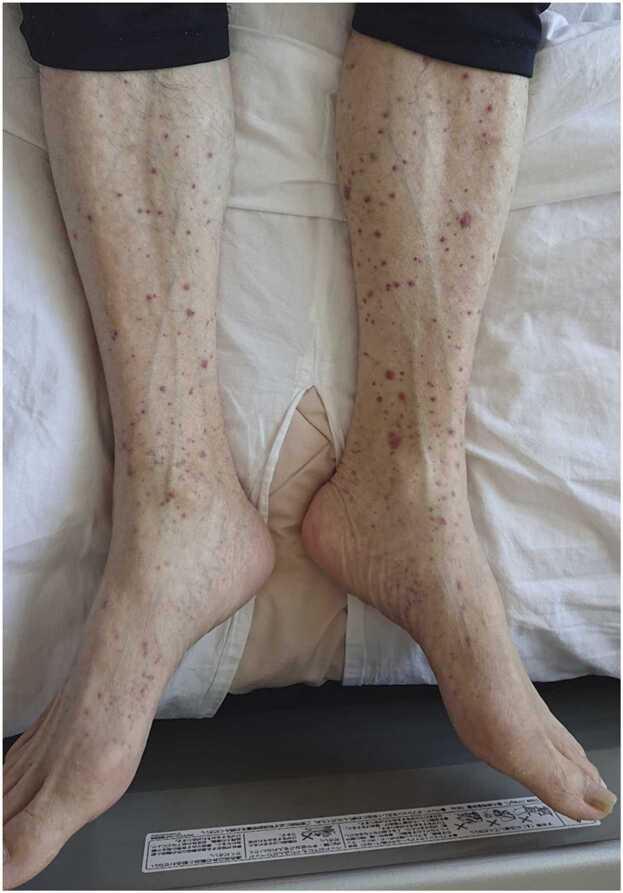
Purpura of the lower leg in a 68-year-old man undergoing IE treatment.

**Fig. 3:**
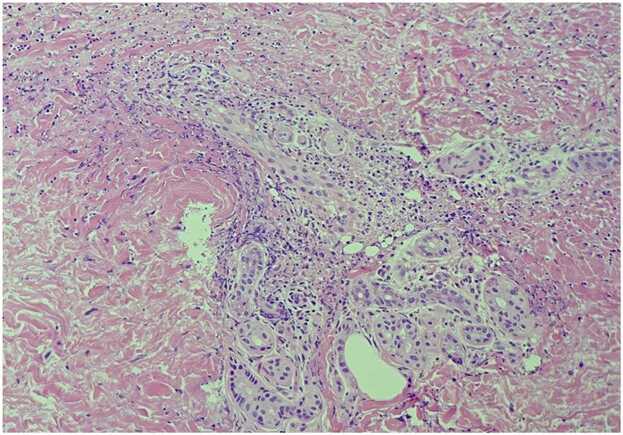
Pathological finding (hematoxylin-eosin stain) of the skin showing cutaneous vasculitis.

A 74-year-old man presented to our hospital complaining of fever. He had a history of chronic heart failure and had previously undergone cardiac resynchronization therapy device (CRT-D) implantation for ventricular tachycardia. Transthoracic echocardiography revealed a 5 × 4 mm vegetation on the aortic valve (Fig. 1). Accordingly, he was diagnosed with infectious endocarditis (IE) and CRT-D infection, and was treated with cefazolin. Methicillin-susceptible *Staphylococcus aureus* was detected on blood culture. On the 15th day of hospitalization, his CRT-D was removed. The following day, purpura appeared on his upper and lower limbs, abdomen, and back (Fig. 2). We suspected immunoglobulin A (IgA) vasculitis, anti-neutrophil cytoplasmic antibody (ANCA)-associated vasculitis, or a drug eruption. Histology of a skin biopsy showed cutaneous vasculitis, without interface dermatitis or eosinophil infiltration (Fig. 3). Although histopathology did not reveal IgA deposits, the patient had abdominal pain, bloody stools, elevated serum creatinine (from 0.83 mg/dL to 1.33 mg/dL), hematuria, and proteinuria, and myeloperoxidase ANCA (MPO-ANCA) and protease 3 ANCA (PR3- ANCA) were both negative. Therefore, the patient met the diagnostic criteria for IgA vasculitis [1]. His symptoms disappeared 2 weeks after starting IE treatment and the deterioration of his renal function stopped. The patient was treated with cefazolin for 8 weeks, after which he was discharged.

IgA vasculitis, which formerly known as Henoch-Schonlein purpurais, is a small vessel vasculitis characterized by purpura, renal dysfunction, abdominal pain, and arthritis. It is associated with infections, autoimmune diseases, and malignancy [2]. IgA vasculitis is more common in children than in adults. The frequency of childhood-onset IgA vasculitis is 3–26 per 100,000 individuals, whereas adult-onset IgA vasculitis occurs in 0.1–1.8 per 100,000 individuals [2]. The present case occurred at the age of 74 years. The likelihood of IgA vasculitis increases when pathology confirms the presence of IgA deposition. The sensitivity of IgA deposition is 81%; therefore, even in the absence of IgA deposition, IgA vasculitis should not be ruled out [2]. In fact, in the present case, no IgA deposition was found. Only about 3.5% of cases of cutaneous vasculitis are associated with IE [3]. Adult-onset IgA vasculitis requires urgent attention as it can progress to end-stage renal failure. When treating patients with IE who develop purpura, clinicians should assess their renal function and ask about gastrointestinal symptoms. If IgA vasculitis is suspected, immediate referral is necessary.

## Ethical approval

The parent’s consent was required for publication.

## Consent

Written informed consent was obtained from the patients for publication of this article.

## Funding

This research did not receive specific grants from public, commercial, or not-for-profit funding agencies.

## CRediT authorship contribution statement

**Kento Furuya**: Data curation, Investigation, Resources, Visualization, Writing – original draft. **Naoya Itoh:** Writing – review & editing.

## Declaration of Competing Interest

The authors have no conflicts of interest to declare.
